# Maternal hyperhomocysteinemia compromises female offspring fertility through overactivation of primordial follicles

**DOI:** 10.1016/j.isci.2026.116393

**Published:** 2026-06-19

**Authors:** Jinmei Gao, Lu Wang, Jie Ma, Jialing Li, Yajie Wang, Jinfang Wang, Rong Hu

**Affiliations:** 1 General Hospital of Ningxia Medical University, Yinchuan, Ningxia, China; 2 Yulin Hospital of the First Affiliated Hospital of Xi’an Jiao Tong University, Yulin, Shanxi, China; 3 Department of Obstetrics, General Hospital of Ningxia Medical University, Yinchuan, Ningxia, China; 4 Reproductive Medicine Center, General Hospital of Ningxia Medical University, Yinchuan, Ningxia, China; 5 Institute of Medical Sciences, General Hospital of Ningxia Medical University, Yinchuan, Ningxia, China

**Keywords:** molecular biology, female reproductive endocrinology, developmental biology

## Abstract

Hyperhomocysteinemia (HHcy), characterized by plasma homocysteine concentrations exceeding 15 μmol/L, has been associated with various issues that impact personal health and offspring well-being. This study examines the effect of maternal HHcy induced by a high-methionine diet on the fertility of female offspring mice. The results showed that maternal HHcy caused the overactivation of primordial follicles in female offspring mice by promoting the phosphorylation of key factors, including RPS6, mTOR, FOXO3a, and AKT. Moreover, the number of mitochondria in mature oocytes decreases, and mitochondrial function decreases, further leading to increased reactive oxygen species (ROS) levels, a higher degree of DNA damage, and spindle abnormalities, ultimately impairing the quality of oocytes. These findings demonstrate that maternal HHcy decreases offspring fertility by inducing primordial follicle overactivation and impairing oocyte quality, providing new insights into the pathological mechanisms through which HHcy affects the reproductive potential of offspring.

## Highlights

• Maternal HHcy adversely affects reproductive health in female offspring
• Maternal HHcy overactivates prepubertal primordial follicles, depleting ovarian reserve early
• Maternal HHcy impairs oocyte quality in offspring

## Graphical abstract

**Figure:**
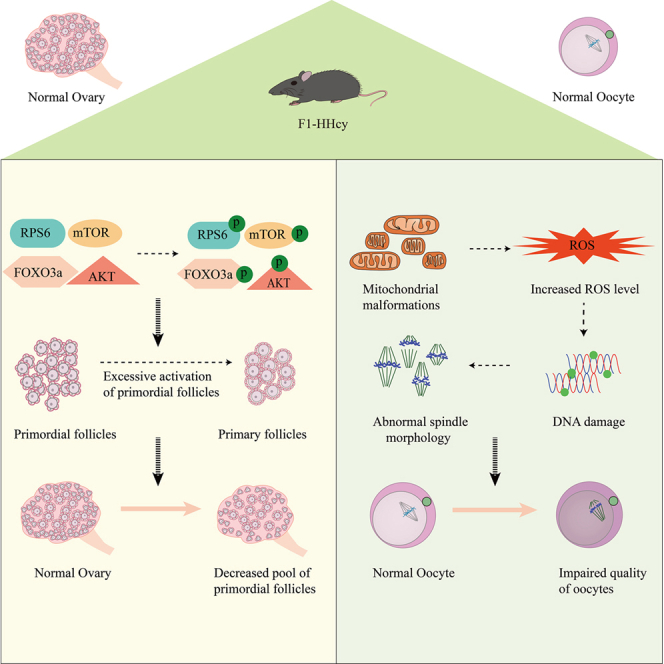

## Introduction

The declining trend in female fertility has become a significant global concern in recent decades. Understanding and addressing the factors behind declining female fertility is critical for supporting women’s reproductive health and ensuring positive outcomes for future generations. Both genetic and environmental factors play important roles in determining offspring growth, development, and metabolic health, according to the developmental origins of health and disease (DOHaD) theory.^1^ Maternal metabolic disorders during pregnancy, such as gestational diabetes, obesity, and hypertension, can significantly impact offspring health, leading to developmental issues and long-term health consequences, the effects of which may persist across multiple generations.^2^^,^^3^^,^^4^^,^^5^^,^^6^^,^^7^^,^^8^ These conditions can also affect reproductive health, potentially causing early or delayed puberty, hormonal imbalances, reduced fertility, and an increased risk of reproductive disorders in both males and females.^9^^,^^10^^,^^11^^,^^12^ Homocysteine (Hcy) is an intermediate in the metabolism of methionine, an essential amino acid. It is either recycled into methionine with the help of vitamin B12 and folic acid or converted to cysteine with the assistance of vitamin B6. The most common causes of elevated Hcy levels include dietary deficiencies, such as inadequate folic acid or vitamin B12 intake, excessive methionine consumption, and genetic mutations in the MTHFR and CBS genes.^13^^,^^14^ Hyperhomocysteinemia (HHcy) is a metabolic disorder characterized by blood Hcy concentrations exceeding 15 μmol/L and is associated with significant health risks.^15^

Recent research has shown that elevated Hcy levels can impair female fertility through abnormal zona pellucida and microvilli formation, increased ovarian atretic follicle formation, and impaired oocyte maturation.^16^ It can also alter the uterine environment, hindering embryo implantation and pregnancy maintenance.^17^ Other evidence suggests that exposure to HHcy early in pregnancy disrupts trophoblast differentiation and has adverse effects on pregnancy outcomes.^18^ Furthermore, HHcy induces oxidative stress, damaging reproductive tissues, which may contribute to fertility issues and conditions such as PCOS.^19^^,^^20^ In addition, maternal HHcy during gestation can negatively impact offspring health, leading to abnormal fetal development, neural tube defects, low birth weight, and other complications.^21^^,^^22^^,^^23^^,^^24^^,^^25^^,^^26^^,^^27^^,^^28^^,^^29^ The effects of maternal HHcy during gestation on follicular development and fertility in female mouse offspring remain poorly understood and are the focus of this study.

The embryonic period and early life are critical for establishing ovarian function in females and are highly susceptible to interference from adverse factors. Taking C57 female mice as an example, by day 17.5 of embryonic development, the syncytium begins to break down as periovarian granulosa cells invade and surround the oocytes, forming primordial follicles. This process completes the establishment of the primordial follicle pool approximately three days after birth. During this time, one-third of the oocytes develop into primordial follicles, while the remaining two-thirds undergo programmed apoptosis.^30^^,^^31^ Before puberty, the hypothalamic pituitary ovarian (HPO) axis is not fully mature, and although many primordial follicles are activated, they do not progress to ovulatory-capable follicles.^32^ Consequently, a substantial loss of primordial follicles occurs, which plays a crucial role in the formation of the ovarian reserve available for reproduction in adulthood. The activation of primordial follicles before puberty is vital for female sexual development and the establishment of the HPO axis.^33^ Disruptions in the formation or activation of primordial follicles can lead to abnormalities in female sexual development, premature ovarian failure, and other complications, significantly affecting female reproductive health.

This study comprehensively assessed the growth, development, and reproductive capacity of female offspring exposed to maternal HHcy and revealed a significant reduction in their reproductive capacity. To elucidate the mechanisms underlying this reduction, the formation and depletion of primordial follicles in offspring mice were tracked. The results revealed that many primordial follicles were activated in the ovaries of the female offspring before puberty, resulting in a diminished ovarian reserve. Additionally, an assessment of oocyte quality in female offspring revealed that maternal HHcy exposure reduced mitochondrial numbers and impaired function, leading to decreased oocyte quality. Therefore, maternal HHcy exposure during gestation impairs the reproductive health of female offspring mice by inducing primordial follicle overactivation and damaging oocyte quality.

## Results

### Maternal HHcy causes decreased survival and ovarian dysfunction in offspring

**Figure 1:**
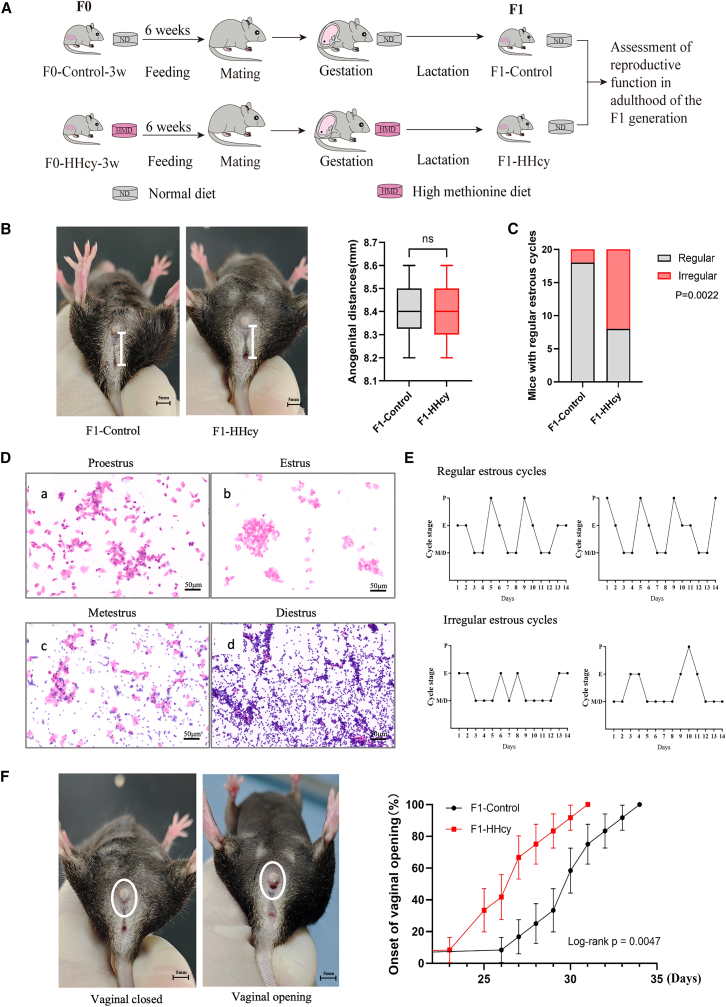
Periconceptional maternal exposure to HHcy causes premature gonadal development and impaired function in F1 female mice (A) Modeling process in F1 female mice. (B) Anogenital distances of female mice in the F1-HHcy and F1-control groups (*n* = 12). The data are presented as boxplots, and statistical analysis was performed using the Mann-Whitney *U* test. Scale bars, 5 mm, ns *p* > 0.05. (C) Percentages of female mice with regular estrous cycles in each group (*n* = 20); the data are presented as percentages (%), and statistical analysis was performed using Fisher’s exact test. ∗*p* < 0.05, ∗∗*p* < 0.01, ∗∗∗*p* < 0.001. (D) Typical morphology of vaginal cells during each estrous cycle, scale bars, 50 μm. (E) Representative graph of 14 consecutive days of estrus cycle monitoring. (F) Onset of vaginal opening in each group (*n* = 12). The data are presented as Kaplan-Meier curves, and statistical analysis was performed using the log rank test. Scale bars, 5 mm, ∗*p* < 0.05, ∗∗*p* < 0.01, ∗∗∗*p* < 0.001.

To investigate the impact of maternal HHcy on the reproductive function of female offspring, first, a female mouse model of HHcy was constructed, as shown in Figure 1A. F0 female mice were fed a high-methionine diet starting at 3 weeks of age for 6 weeks. Serum Hcy levels were measured and exceeded the standard threshold of 15 μmol/L, confirming successful modeling (Figure S1A). This method was subsequently used for subsequent experiments. To maintain serum Hcy levels during gestation and lactation, the high-methionine diet was continued throughout gestation and lactation until the offspring were weaned and then replaced with a normal diet. The number of offspring born on the day of birth and the number of offspring still alive on day 7 were recorded. The survival rate of the offspring was subsequently calculated, after which all the male offspring were eliminated. The results revealed that the survival rate of mice in the F1-HHcy group was significantly reduced (Figure S1B). To assess the growth and development of female offspring mice, weekly monitoring and statistical analysis of the body weight gain in F1 females revealed that the body weight of F1-HHcy females was significantly lower than that of the F1-control group (Figure S1C). Serum Hcy levels were measured at 8 weeks of age, ruling out any potential health effects of abnormal Hcy levels in F1 mice. The serum Hcy levels in female mice from both groups were within the normal range (Figure S1D). To assess the health status of the female offspring mice, tissue sections from the heart, liver, spleen, lungs, and kidneys of the two groups of mice were stained, and ELISA kits were used to test indicators related to liver and kidney function. No significant abnormalities were detected in the results of the histomorphological examinations or liver and kidney function tests (Figures S1E and S1F). The above results suggest that although mice in the F1-HHcy group had reduced survival rates and slow weight gain, their vital organ and organ functions were not significantly impaired.

**Figure 2:**
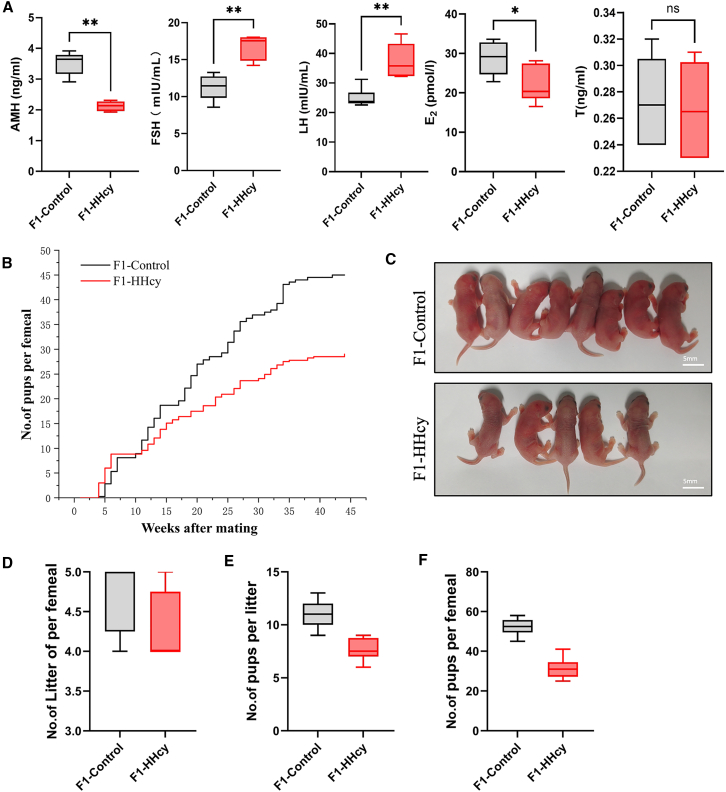
Maternal perinatal exposure to HHcy results in decreased fertility in F1 female mice (A) Serum levels of sex hormones in female F1 mice at 12 weeks (*n* = 6). (B) Litter size of F1 female mice for 10 consecutive months. (C) Representative images per litter of F1 female mice (*n* = 12), scale bars, 5 mm. (D) The average number of litters per F1 female mouse (*n* = 12). (E) The average number of pups per litter per F1 female mouse (*n* = 12). (F) The total number of litters per F1 female mouse (*n* = 12). The data are presented as boxplots, and statistical analysis was performed using the Mann-Whitney *U* test. ns *p* > 0.05, ∗*p* < 0.05, ∗∗*p* < 0.01, ∗∗∗*p* < 0.001.

This study focuses on the development of the reproductive system in female offspring, and the anogenital distance can be used as a phenotypic trait to understand the influence of genetic factors on the development of the reproductive system. Accordingly, the anogenital distance was measured at 3 weeks of age, and there was no significant difference between the two groups (Figure 1B). The time at which vaginal opening occurs in female mice is an important indicator of sexual maturity. These findings help us to understand the status of hormone secretion in mice and the influence of genetic factors on sexual maturity. The timing of vaginal opening in the offspring was continuously monitored, and the results revealed that compared with F1-control females, F1-HHcy females exhibited earlier vaginal opening (Figure 1F). At 10 weeks of age, the estrous cycle was monitored for 14 consecutive days to assess ovarian function in adult F1 females. The results indicated a higher prevalence of estrous cycle disorders in F1-HHcy females (Figures 1C–1E). Serum sex hormone levels were also measured after estrous cycle monitoring. While testosterone (T) levels did not significantly change, follicle-stimulating hormone (FSH) and luteinizing hormone (LH) levels significantly increased, whereas anti-Müllerian hormone (AMH) and estradiol (E2) levels significantly decreased (Figure 2A).

### Maternal HHcy leads to decreased fertility in female offspring

Ovarian function is a key determinant of female fertility, with follicles serving as the fundamental units that sustain it. During puberty, the ovaries begin normal functioning, and the number of follicles present at this stage directly influences fertility and can serve as an indicator of the onset of ovarian failure. Mili-Vasa Homologue (MVH) is specifically expressed in primordial germ cells (PGCs) and oocytes, thereby allowing the tracking of oocyte development, number, and location. In this study, ovarian tissue sections from Pd21 offspring mice were used for MVH histochemistry and immunofluorescence staining to evaluate the ovarian reserve of adolescent mice. Taken together, the results revealed that the number of MVH-positive cells in the F1-HHcy group was lower than that in the F1-control group (Figures S2A and S2B). Moreover, ovulation promotion experiments on 8-week-old F1 females revealed that the F1-HHcy group produced significantly fewer oocytes than the F1-control group did (Figure S2C).

Breeding experiments are among the most authoritative methods for assessing female fertility. Analysis of the litter size of F1 female mice over 10 consecutive months revealed that compared with F1-control females, F1-HHcy females had significantly smaller litters and stopped giving birth earlier (Figure 2B). Representative graphs were also generated to illustrate the litter size and production for females in both the F1-control and F1-HHcy groups (Figure 2C). Statistical analysis of the total number of litters, average number of pups per litter, and number of litters per female revealed that all these parameters were significantly lower in the F1-HHcy females than in the F1-control group (Figures 2D–2F).

### Maternal HHcy did not affect the establishment of primordial follicles in offspring

**Figure 3:**
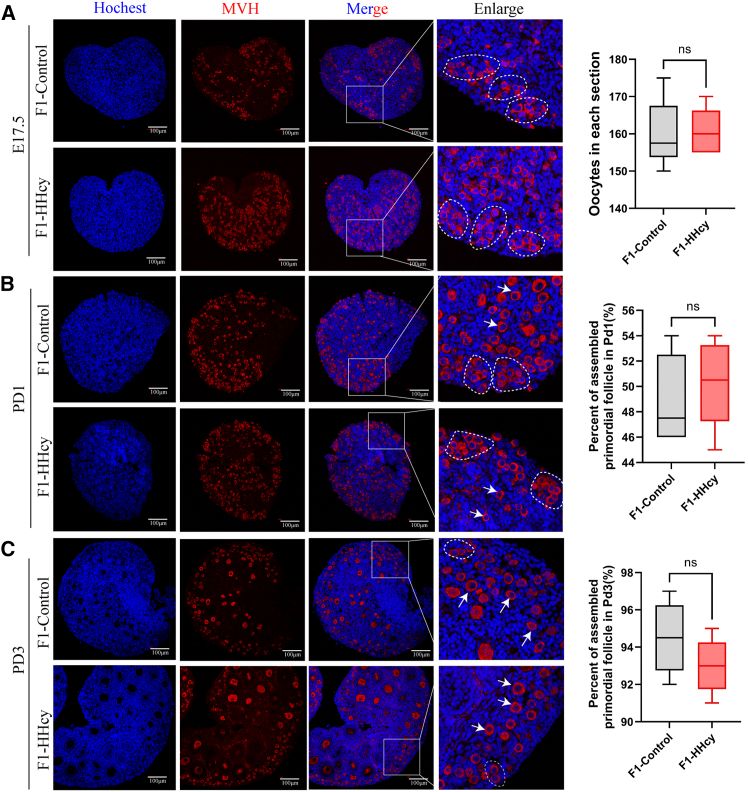
Periconceptional maternal exposure to HHcy did not affect the formation of primordial follicles in F1 female mice (A) Immunofluorescence staining of the ovarian tissue at E17.5 in F1 females and the number of oocytes per section (*n* = 6), scale bars, 100 μm. (B) Immunofluorescence staining of ovarian tissue at Pd1 and percentage of oocytes within cysts or follicles in F1 females (*n* = 6), scale bars, 100 μm. (C) Immunofluorescence staining of ovarian tissue at Pd3 and percentage of oocytes within cysts or follicles in F1 females (*n* = 6), scale bars, 100 μm. The data are presented as boxplots, and statistical analysis was performed using the Mann-Whitney *U* test. ns *p* > 0.05, ∗*p* < 0.05, ∗∗*p* < 0.01, ∗∗∗*p* < 0.001.

The formation of primordial follicles, which is crucial for the development of a normal ovarian reserve, occurs primarily in female mice during the embryonic and early postnatal periods. On embryonic day 17.5, germ cells exist primarily in the form of germ cysts. These disaggregate within approximately 3 days after birth, resulting in the formation of primordial follicles. The process of cyst depolymerization determines the number of germ cells that ultimately contribute to the ovarian reserve. In this study, MVH immunofluorescence staining was performed on ovarian tissue from embryos at Pd17.5, Pd1, and Pd3 to quantify the number of germ cells and evaluate the process of cyst depolymerization. The total number of oocytes in the ovarian sections was counted in 5 consecutive sections, with germ cells containing more than 2 connected oocytes considered cystic germ cells and single unconnected germ cells categorized as follicular germ cells. The ratio of cystic/follicular germ cells to total germ cells was used to calculate the percentage of follicles among the germ cells (Figures 3A–3C). The results showed that the formation of primordial follicles was not significantly affected in F1-HHcy female mice.

### Maternal HHcy leads to a substantial loss of prepubertal primordial follicles in offspring

**Figure 4:**
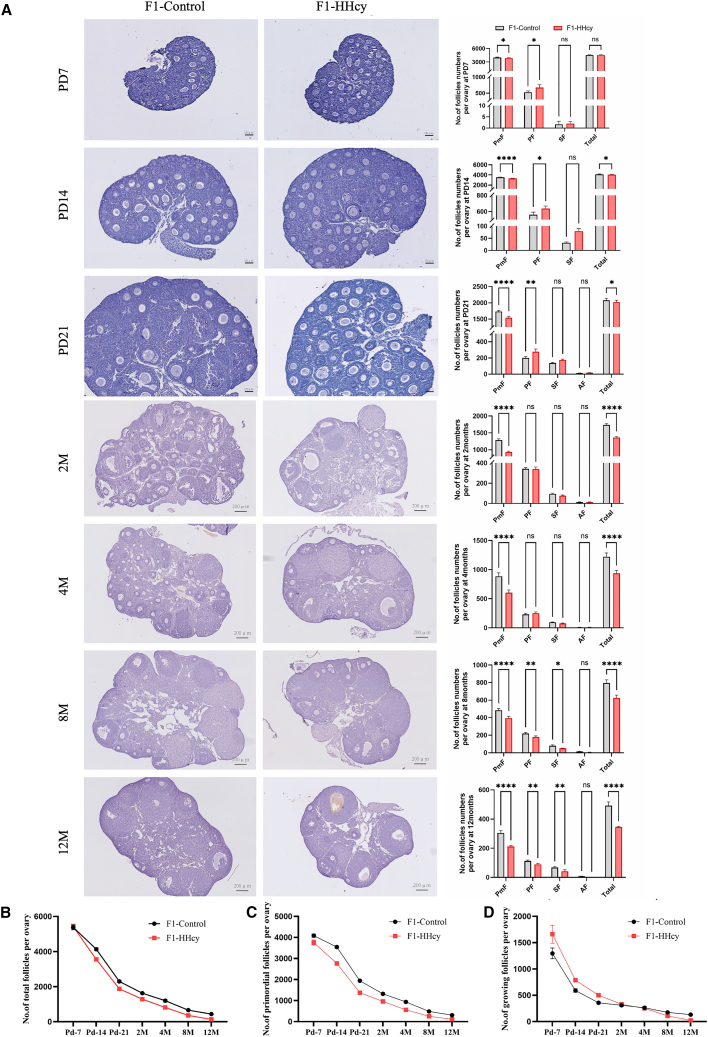
Periconceptional maternal exposure to HHcy accelerates primordial follicular depletion in prepubertal F1 female mice (A) Hematoxylin staining of ovarian tissue at different time points and statistical analysis of follicle counts at all time points (*n* = 6). The data are presented as boxplots, and statistical analysis was performed using the Mann-Whitney *U* test. Scale bars, 100 μm or 200 μm, ns *p* > 0.05, ∗*p* < 0.05, ∗∗*p* < 0.01,∗∗∗*p* < 0.001, ∗∗∗∗*p* < 0.0001. (B) Line graph of the change in total follicle count. (C) Line graph of the change in primordial follicle count. (D) Line graph of the change in the number of growing follicles.

The rate of depletion of primordial follicles determines a woman’s reproductive lifespan. Therefore, in this study, the entire process of primordial follicle depletion in the ovaries was tracked. Successive hematoxylin staining of ovarian tissue sections from offspring at Pd7, Pd14, Pd21, 2 M, 4 M, 8 M, and 12 M was performed to determine the changes in the number of follicles at all time points to observe the process of germ cell depletion. Both the number of primordial follicles and the total number of follicles were significantly lower in the ovaries of the F1-HHcy group than in those of the F1-control group in adulthood (Figure 4A). In the present study, line graphs were used to observe the depletion of total, primordial, and growing follicles (including primary, secondary, and antral follicles) in the ovaries of F1 females. The results revealed that the total number of follicles was depleted earlier in the F1-HHcy group than in the F1-control group (Figure 4B). Observations of changes in the number of primordial follicles revealed a drastic reduction in primordial follicles in the F1-HHcy group compared with the F1-control group before puberty (Pd7-Pd21) (Figure 4C). Changes in the number of growing follicles also reflected significantly more growing follicles in the F1-HHcy group than in the F1-control group before puberty (Figure 4D).

### RNA-seq reveals changes in the ovarian tissue of prepubertal offspring

**Figure 5:**
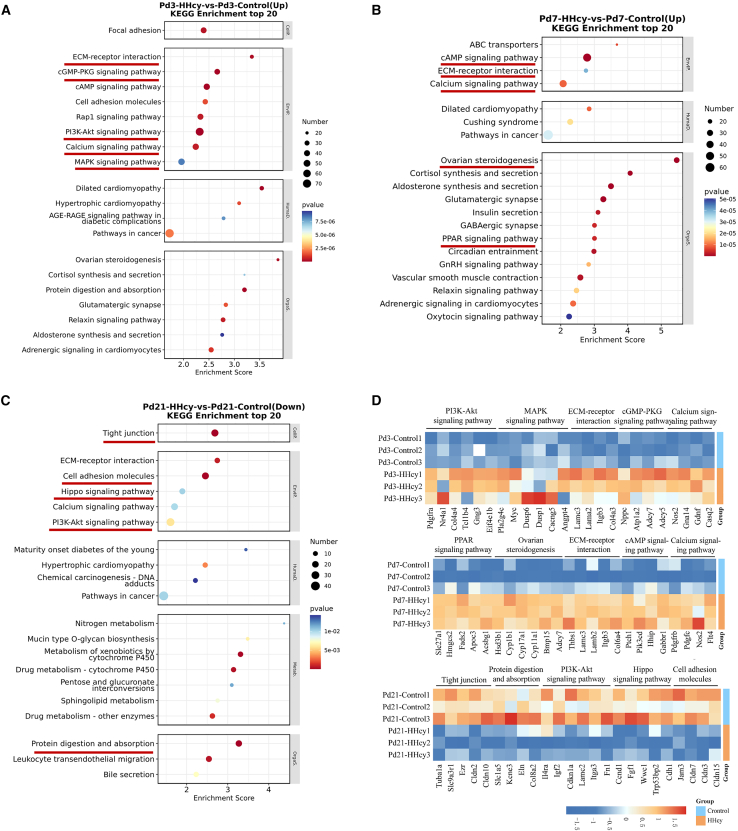
Maternal perinatal exposure to HHcy alters gene transcript levels in prepubertal F1 female ovarian tissue (A) KEGG gene enrichment analysis of F1 female ovarian tissue on Pd3 revealed upregulated signaling pathways. (B) KEGG gene enrichment analysis of F1 female ovarian tissue on Pd7 revealed upregulated signaling pathways. (C) KEGG gene enrichment analysis of F1 female ovarian tissue on Pd21 mice revealed downregulated signaling pathways. (D) The expression levels of the key genes in the significantly different signaling pathways shown in the KEGG enrichment analysis are displayed in heatmaps.

The period before puberty is critical for ovarian development and is susceptible to various factors that ultimately affect ovarian function. Previous investigations have suggested that the main reason for the decline in fertility in the F1-HHcy group was the overactivation and subsequent loss of primordial follicles in ovarian tissue before puberty. To elucidate the underlying mechanisms, the present study used ovarian tissue from F1 females at Pd3, Pd7, and Pd21 for transcriptome sequencing. Strong within-group consistency was demonstrated by transcriptome sequencing for principal-component analysis (PCA) (Figures S3A, S4A, and S5A), with genes significantly upregulated in the ovarian tissue of the F1-HHcy group at Pd3 and Pd7 (Figures S3B and S4B) and significantly downregulated at Pd21 (Figure S5B). Gene Ontology (GO) enrichment analysis revealed that these DEGs are associated primarily with cellular components, especially those related to the extracellular matrix (ECM) (Figures S3C, S4C, and S5C). Kyoto Encyclopedia of Genes and Genomes (KEGG) analysis revealed significant upregulation of pathways involved in primordial follicle activation, such as phosphatidylinositol 3-kinase (PI3K)-protein kinase B (AKT), mitogen-activated protein kinase (MAPK), ECM-receptor interaction, calcium signaling, cyclic AMP (cAMP), ovarian steroidogenesis, and PPAR at Pd3 and Pd7, in F1-HHcy (Figures 5A and 5B). In contrast, at Pd21, pathways such as tight junctions, cell adhesion molecules, Hippo, PI3K-AKT, and protein digestion and absorption were enriched among significantly downregulated genes (Figure 5C). Heatmap visualization of key gene expression changes provides an intuitive comparison of gene expression levels between the F1-control and F1-HHcy groups (Figure 5D). The sequencing results, which are consistent with previous counts of ovarian tissue sections, support the idea that the overactivation and loss of primordial follicles in the F1-HHcy group occur on Pd3 and Pd7 to such an extent that the ovarian reserve is markedly diminished on Pd 21.

### Mechanisms of overactivation of primordial follicles in prepubertal offspring

**Figure 6:**
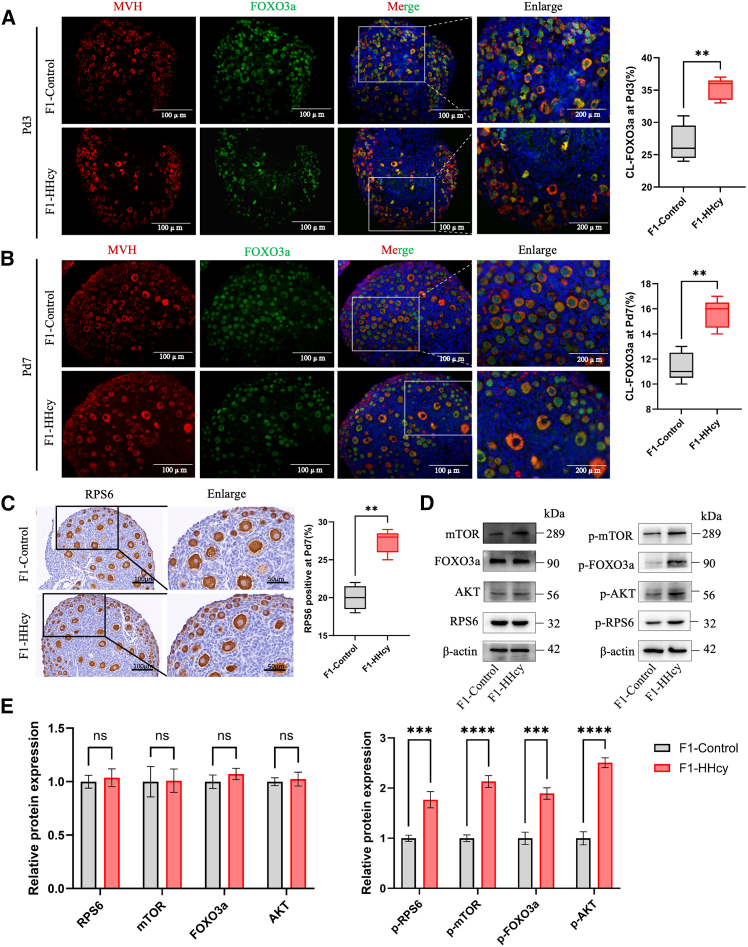
Periconceptional maternal exposure to HHcy promotes primordial follicular mass activation in prepubertal F1 female mice (A) Immunofluorescence staining for MVH (red) and FOXO3a (green) in ovarian tissue from Pd3 F1 and quantification of the proportion of cytoplasmic FOXO3a-positive cells (*n* = 3), scale bars, 100 μm. (B) Immunofluorescence staining for MVH (red) and FOXO3a (green) in ovarian tissue from Pd3 F1 and quantification of the proportion of cytoplasmic FOXO3a-positive cells (*n* = 3), scale bars, 100 μm. (C) Immunohistochemical staining for RPS6 in ovarian tissue sections from Pd7 F1 mice and quantification of the proportion of RPS6-positive cells (*n* = 3), scale bars, 50 μm. (D) Immunoblotting of the primitive follicle activation-associated proteins RPS6, mTOR, FOXO3a, and AKT and their phosphorylated forms *p*-RPS6, *p*-mTOR, *p*-FOXO3a, and *p*-AKT in Pd7 ovarian tissue. (E) Results from the quantitative analysis of RPS6, mTOR, FOXO3a, and AKT protein levels and their phosphorylation levels (*n* = 3). The data are presented as boxplots, and statistical analysis was performed using the Mann-Whitney *U* test. ns *p* > 0.05, ∗*p* < 0.05, ∗∗*p* < 0.01, ∗∗∗*p* < 0.001, ∗∗∗∗*p* < 0.0001.

To further validate the molecular mechanisms of primordial follicle activation, this study selected and validated key molecules involved in this process, namely, ribosomal protein S6 (RPS6), mTOR, Forkhead box O3 (FOXO3a), and AKT, by immunofluorescence, immunohistochemistry, and protein blotting experiments. FOXO3a was localized to the nucleus of dormant primordial follicles, which were maintained in a dormant state through the inhibition of the expression of cell cycle proteins. We quantified the percentage of FOXO3a nucleation by immunofluorescence staining at Pd3 and Pd7, and this value was significantly greater in the F1-HHcy group than in the F1-control group (Figures 6A and 6B). Once primordial follicle activation occurs, RPS6 is phosphorylated by S6 kinase (S6K) in a mechanistic manner, increasing the translational efficiency of ribosomes and promoting the synthesis of specific proteins essential for cell proliferation and primordial follicle activation. Immunohistochemical results revealed that the proportion of RPS6-positive cells in ovarian tissue was significantly greater in the F1-HHcy group than in the F1-control group (Figure 6C).

The AKT and mTOR signaling pathways are highly involved in the activation of primordial follicles. When AKT is activated to a phosphorylated form, it can directly or indirectly regulate the activity of several downstream proteins, such as by promoting FOXO3a phosphorylation and intranuclear translocation to the cytoplasm, which can lead to follicle activation from the dormant state. Moreover, AKT can further regulate follicle development by phosphorylating mTOR, which serves as an important nutrient and energy sensor, and its activation promotes follicle activation and growth. Protein blotting revealed no significant changes in the protein levels of RPS6, mTOR, FOXO3a, and AKT in ovarian tissue samples from Pd7; however, the phosphorylation levels of these proteins significantly increased (Figures 6D and 6E). These results provide further evidence of the overactivation of primordial follicles in the prepubertal ovarian tissue of the F1-HHcy group.

### Maternal HHcy causes impaired oocyte quality in offspring

**Figure 7:**
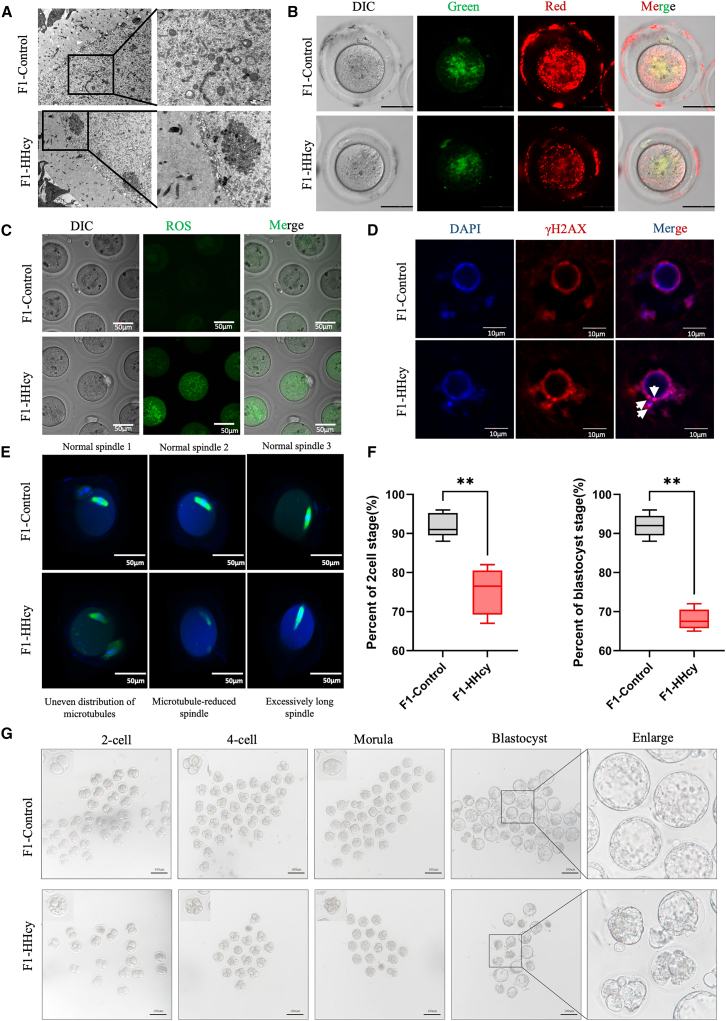
Periconceptional maternal exposure to HHcy impairs oocyte quality and embryonic development in F1 female mice (A) Mitochondria in F1 female mouse oocytes observed by transmission electron microscopy, scale bars, 2 μm or 1 μm. (B) Mitochondrial function in F1 female mouse oocytes determined using JC-1 staining, scale bars, 20 μm. (C) Assessment of ROS levels in the oocytes of female F1 mice, scale bars, 50 μm. (D) Immunofluorescence staining for γH2AX to assess DNA damage in oocytes from F1 female mice, scale bars, 10 μm. (E) Spindle morphology in the oocytes of female F1 mice; microtubules (alpha-tubulin, green); chromatin (hoechst, blue), scale bars, 50 μm. (F) Quantification of 2-cell and blastocyst formation rates following *in vitro* fertilization of F1 female mice (*n* = 6). The data are presented as boxplots, and statistical analysis was performed using the Mann-Whitney *U* test. ns *p* > 0.05, ∗*p* < 0.05, ∗∗*p* < 0.01. (G) Representative images of two-cell, four-cell, mulberry, and blastocyst embryos after *in vitro* fertilization of F1 female mice, scale bars, 100 μm.

In addition to oocyte number, oocyte quality also significantly affects a woman’s fertility. The mitochondrial content and function in oocytes are crucial for energy production, metabolism, oocyte quality, and early embryonic development. In this study, mitochondrial changes in ovarian tissues were examined using transmission electron microscopy. In the F1-control group, the mitochondria in the oocytes appeared regular in morphology and intact in structure. In contrast, oocytes from the F1-HHcy group exhibited a significant reduction in the number of mitochondria, with many showing vacuolization and aggregation with other abnormal organelles (Figures 7A and S6A). The mitochondrial membrane potential is a key indicator of mitochondrial function and is assessed using JC-1 dye. JC-1 can penetrate the cell membrane and either accumulate or disperse within the cell on the basis of the mitochondrial membrane potential. Under normal conditions, JC-1 aggregates in the mitochondria and emits red fluorescence. However, when the mitochondrial membrane potential decreases, such as during early apoptosis or cellular injury, JC-1 remains in its monomeric form and emits green fluorescence.^34^ The mitochondrial membrane potential of the oocytes in the F1-HHcy group was significantly lower than that in the F1-control group (Figures 7B and S6B).

Mitochondrial dysfunction is known to lead to an increase in reactive oxygen species (ROS) levels through various mechanisms. Excessive ROS levels can damage cellular structures and impair function, ultimately compromising oocyte health and viability. Under normal conditions, cells maintain redox homeostasis by neutralizing these free radicals through antioxidant systems such as superoxide dismutase and glutathione. However, when mitochondrial function is impaired or external stressors are heightened, ROS production can exceed the capacity of these antioxidant defenses. In this study, compared with those from the F1-control group, the ROS levels from the F1-HHcy group were significantly greater (Figures 7C and S6C). Elevated ROS levels can cause DNA damage, including base oxidation, strand breaks, and other alterations, which compromise the stability and function of DNA. Maintaining DNA integrity in oocytes is crucial for preserving genetic information and ensuring healthy embryonic development. γH2AX, the phosphorylated form of histone H2AX, serves as a marker of DNA damage. Upon DNA damage, H2AX is rapidly phosphorylated to form γH2AX at damage sites, resulting in distinct phosphorylation foci. Compared with those from the F1-control group, oocytes from the F1-HHcy group showed significantly greater DNA damage (Figures 7D and S7D).

When DNA damage occurs, cells activate checkpoint mechanisms to repair it. However, if the damage is too severe to repair, it may disrupt spindle assembly and function. The spindle in oocytes is composed of microtubules, microtubule-associated proteins, kinesins, and other components, forming a highly organized and dynamic structure essential for proper chromosome segregation during cell division. The integrity and function of the spindle are critical for oocyte health and successful embryonic development. In this study, we assessed oocyte spindle morphology using laser confocal continuous scanning and three-dimensional reconstruction. Compared with the F1-control group, the F1-HHcy group had a significantly greater proportion of abnormal spindles (Figures 7E and S6E). The early development of embryos in *in vitro* fertilization (IVF) experiments can indirectly reflect oocyte health in terms of chromosome integrity, cell division capacity, metabolic status, etc. We evaluated embryo development post-fertilization and revealed that the 2-cell and blastocyst formation rates were significantly decreased in the F1-HHcy group (Figures 7F and 7G).

## Discussion

The earlier vaginal opening observed in F1-HHcy females (Figure 1C) suggests that maternal HHcy may disrupt the precise hormonal regulation governing pubertal onset in female offspring. These findings point to a potential programming effect of HHcy on the developing neuroendocrine axis, which could have implications for long-term reproductive health and warrants further investigation into the underlying molecular mechanisms. Endocrine analysis (Figure 1G) corroborated the observed reproductive phenotype. The concurrent increase in FSH/LH and reduction in AMH/E2 are indicative of compromised ovarian function and disrupted HPG axis feedback. These findings, together with the estrous cycle abnormalities, demonstrate that maternal HHcy leads to lasting impairments in the hormonal regulation and reproductive capacity of female offspring. Longitudinal breeding data over 10 months revealed that compared with control females, F1-HHcy females exhibited significantly reduced litter sizes and earlier reproductive cessation (Figure 2). These functional impairments, as evidenced by decreases in total litters, number of pups per litter, and reproductive lifespan, demonstrate that maternal HHcy accelerates ovarian aging and compromises fertility in female offspring.

Correct establishment and orderly depletion of the primordial follicle pool are important determinants of reproductive longevity and fertility.^33^^,^^35^^,^^36^ Longitudinal follicle counts revealed that maternal HHcy accelerated the loss of ovarian reserve through the prepubertal overactivation of primordial follicles. The premature depletion of primordial follicles and subsequent surge in growing follicles (Figure 4) establish a direct link between developmental HHcy exposure and premature ovarian aging. This mechanistic insight explains the concurrent hormonal imbalances and fertility decline observed in F1-HHcy females. To investigate the causes of reduced fertility in F1-HHcy female mice further, immunofluorescence staining for MVH and FOXO3a was used to analyze the formation process of primordial follicles in mice from embryonic day 17.5 to postnatal day 3, and HE staining was used to analyze the depletion process in mice from postnatal day 7–12 months of age (Figures 3 and 4 ). Substantial experimental verification confirmed that female F1-HHcy mice experience a significant loss of primordial follicles from Pd3 to Pd21 (Figures 5 and 6); moreover, oocyte quality is impaired (Figure 7). Both of these changes are primary factors contributing to their reduced fertility.

Previous studies have shown that maternal HHcy during the gestation period contributes to suboptimal nutrient and oxygen delivery to the fetus by compromising vascular health and disrupting placental perfusion.^28^ Perinatal hypoxia can lead to the activation of primordial follicles and premature depletion of the ovarian reserve.^27^^,^^37^^,^^38^ Simultaneously, the hypoxic environment caused by HHcy activates hypoxia-inducible factors, induces endothelial dysfunction, and promotes the production of inflammatory mediators, which together drive and amplify inflammation. This not only impairs fetal development but also increases the risk of chronic conditions such as cardiovascular and metabolic disorders.^27^ Both hypoxia and inflammation can activate the PI3K/AKT signaling pathway, which in turn stimulates mTOR activity. The activation of this pathway in response to oxidative stress, cytokine signaling, and altered oxygen levels can push dormant primordial follicles into the growing pool. The levels of RPS6, mTOR, FOXO3a, and AKT, key signaling molecules and effectors involved in primordial follicle activation, significantly increased, indicating that massive activation of primordial follicles occurred in the ovary (Figures 6D and 6E). Moreover, maternal HHcy increases oxidative stress, which can damage cellular structures, including DNA, proteins, and lipids, disrupting normal cellular functions, and potentially affecting embryonic development.^29^ Furthermore, Hcy plays a role in atherogenesis and cognitive impairment through the regulation of epigenetic modification.^39^^,^^40^ Yakovleva et al. demonstrated that maternal HHcy induces transgenerational cognitive and behavioral deficits in second-generation offspring through epigenetic mechanisms.^41^ Epigenetic regulation is critical for the maturation of the oocyte and the formation of the syncytium during reproduction, as well as for the formation of primordial follicles during the embryonic stage.^42^^,^^43^^,^^44^ Significantly reduced levels of H3K4 methylation in the oocytes of aged mice decreased transcriptional activity in the oocytes and increased DNA methylation, which impaired the developmental potential of the oocytes and the fertility of female mice.^45^ The combined effects of these factors can lead to long-term reproductive dysfunction and reduced female fertility in offspring.^46^

Analysis of RNA sequencing (RNA-seq) results from the ovaries of offspring revealed a significant increase in the number of genes upregulated at Pd3 and Pd7, suggesting active metabolism in ovarian tissue. KEGG analysis of the upregulated genes revealed enrichment in signaling pathways such as ECM receptors, calcium signaling, and ovarian steroidogenesis. ECM receptors on oocytes and granulosa cells can influence the activation of primordial follicles by modulating the signaling pathways that control cell proliferation and survival.^47^ Calcium signaling in granulosa cells can trigger the transition of primordial follicles into primary follicles.^48^ Steroidogenesis influences primordial follicle activation by modulating the hormonal environment that regulates follicular growth. The balance of steroid hormones is influenced by signaling pathways, including those involving ECM receptors and calcium.^49^ The sequencing results, together with previous observations of ovarian tissue sections, suggest that many primordial follicles become active in the ovaries of female F1-HHcy mice before puberty. This finding also reveals the cause of precocious puberty in the F1-HHcy group.

However, at the Pd21 stage, the number of downregulated genes in the ovaries increased significantly, suggesting a lower metabolic level in ovarian tissue. KEGG analysis of the downregulated genes revealed that signaling pathways associated with primordial follicle activation, including tight junction, the PI3K-AKT pathway, cell adhesion molecules, protein digestion, and absorption, were significantly enriched, which can significantly impact ovarian reserve. Once tight junctions are downregulated, communication between granulosa cells and the oocyte can be disrupted. This disruption might compromise the nutrient and hormonal signaling necessary for oocyte maturation and health, potentially reducing the ovarian reserve. Additionally, impaired tight junctions could lead to increased susceptibility to oxidative stress and inflammation, further impacting ovarian function and reducing the number of viable oocytes.^50^ Downregulation of PI3K-AKT signaling can impair the activation of primordial follicles, leading to a decreased rate of follicle recruitment. This reduces the overall number of follicles that are available for maturation, thus depleting the ovarian reserve over time.^51^ The downregulation of cell adhesion molecules could weaken the connection between the oocyte and granulosa cells or theca cells. This could impair follicular development and reduce the ability of oocytes to survive and mature. Furthermore, altered cell adhesion can interfere with the synchronization of follicular growth, leading to reduced oocyte quality and quantity over time.^52^ The downregulation of protein digestion and absorption could result in a shortage of critical nutrients for ovarian cells, impairing their function. This could affect the development of ovarian follicles and lead to premature follicular atresia. As a result, the ovarian reserve may decrease, and the quality of the oocytes could be compromised, reducing their fertility potential.^53^ In summary, the downregulation of these signaling pathways can disrupt the ovarian microenvironment, affecting both the quantity and quality of oocytes. During this period, primordial follicle reserves are established in the ovaries, the hormone-synthesizing system develops, the HPO axis is formed, and the follicles begin to mature.^32^^,^^54^^,^^55^ These processes lay the foundation for the maturation of ovarian function, hormonal fluctuations, and the normal progression of the menstrual cycle after puberty. This explains the disruption of the estrous cycle and hormone levels in the F1-HHcy group (Figures 1D–1G).

The ovaries are the first organs to age in females, and they are susceptible to adverse internal and external influences. Adverse effects in prepubertal mice impair oocyte quality and function in adulthood.^56^ In particular, reduced ovarian reserve in adult female mice has a negative effect on oocyte quality.^57^ Mechanisms include reduced levels of key metabolites that protect oocytes against aging, such as spermine, in ovarian tissue^58^ and increased iron accumulation in the ovarian microenvironment,^34^ which affects mitochondrial function and energy metabolism and disrupts the internal redox balance in ovarian tissue, ultimately compromising oocyte quality. Sequencing of ovarian tissue on Pd 21 revealed inhibition of the PI3K/AKT pathway in the F1-HHcy group (Figure 5C), which is essential for maintaining oocyte quality.^59^ Moreover, a critical event in the primordial-to-primary follicle transition is the differentiation of flattened pregranulosa cells into cuboidal, functionally active granulosa cells, a process demonstrated to be directly driven by mTORC1 signaling within the oocyte and surrounding somatic cells.^60^^,^^61^ Therefore, the observed alteration in phosphorylated mTOR (*p*-mTOR) levels in our model could indicate impaired signaling for this essential cellular transition. Consequently, while our current data reveal a net reduction in the primordial follicle pool, it remains to be determined whether this is primarily due to accelerated but aberrant activation, in which follicles initiate growth but fail because of inadequate granulosa cell support, or suppression of activation, leading to a dormant but ultimately atretic reserve.

First, the process of overactivation itself may not be synchronous or perfectly coordinated. Follicles entering the growth pool under metabolic stress may have intrinsic defects in oocyte-somatic cell communication from the outset. This could lead to suboptimal granulosa cell differentiation and function, as discussed regarding mTOR signaling, resulting in a compromised follicular microenvironment that fails to support full oocyte competency during its prolonged growth phase. Second, the concept of the “production line” hypothesis or functional heterogeneity within the primordial follicle pool is relevant. It is possible that HHcy-induced signaling dysregulation does not selectively activate follicles but rather disrupts the normal, staggered recruitment process. This could lead to the premature activation and expenditure of a developmentally privileged subpopulation of oocytes, resulting in the formation of a lower-quality cohort for later reproductive life. Alternatively, the stress of overactivation may induce epigenetic or metabolic alterations in the oocyte genome that are propagated through folliculogenesis, ultimately manifesting as reduced embryonic developmental potential post ovulation. In summary, while our study revealed a strong link between maternal HHcy, primordial follicle overactivation, and reduced fecundity, the precise cellular and molecular pathways leading from dysregulated early recruitment to poor oocyte quality remain critical areas for future study.

We assessed mitochondrial function in oocytes using JC-1 dye-labeled mitochondria and observed decreased mitochondrial activity (Figure 7B). Cytosolic ROS were once considered mere byproducts of mitochondrial oxygen metabolism; now, they are also known to participate in various signaling pathways, and for example, they significantly increase DNA damage.^62^ The spindle is a crucial structure responsible for chromosome segregation during meiosis. Abnormal spindle morphology disrupts the proper attachment of chromosomes to spindle microtubules, leading to chromosome mis-segregation or DNA damage, which significantly reduces oocyte quality and embryonic developmental potential.^63^ The compromised oocyte quality in F1-HHcy females was evidenced by both structural and functional deficits. The increased proportion of abnormal meiotic spindles (Figures 7E and S6E) suggests that maternal HHcy disrupts the dynamic organization of microtubules, which is essential for accurate chromosome segregation. This structural impairment translated into reduced developmental competence, as demonstrated by the decreased 2-cell and blastocyst formation rates following IVF (Figures 7F and 7G). Collectively, these findings indicate that maternal HHcy compromises offspring fertility through the following dual mechanisms: prepubertal depletion of ovarian reserve via primordial follicle overactivation and impaired oocyte quality resulting from mitochondrial dysfunction and redox imbalance.

**Figure 8:**
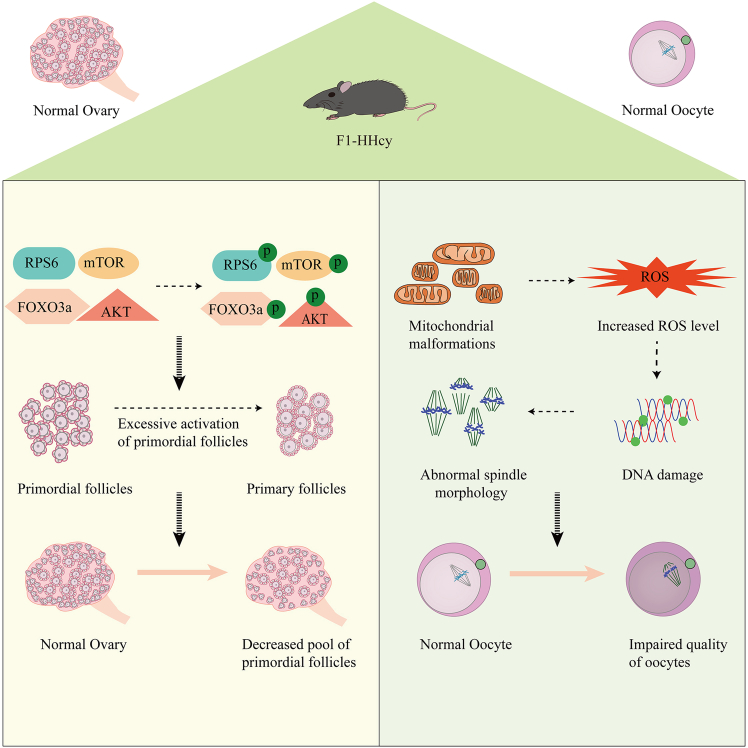
Description of the mechanisms by which maternal periconceptional HHcy exposure impairs F1 female mouse reproductive function Increased phosphorylation of factors such as RPS6, mTOR, FOXO3a, and AKT in the ovarian tissue of F1-HHcy female mice before puberty promotes massive activation and loss of primordial follicles, leading to a reduction in ovarian reserve. Moreover, the number of mitochondria in the oocyte is reduced, and their function is impaired, which further leads to increased levels of ROS, increased levels of DNA damage, and spindle abnormalities in the oocyte, impairing oocyte quality and embryo developmental potential and ultimately reducing the fertility of adult offspring.

This study revealed that maternal HHcy during the gestation period impairs fertility in female offspring through two primary mechanisms (Figure 8). First, maternal HHcy increases the phosphorylation of key follicular activation factors (e.g., RPS6, mTOR, FOXO3a, and AKT) in the prepubertal ovary, leading to the overactivation of primordial follicles and a subsequent reduction in ovarian reserve. Second, it compromises oocyte quality and embryo development by disrupting mitochondrial function, increasing ROS levels, and inducing DNA damage and spindle abnormalities. These findings highlight the importance of monitoring maternal Hcy levels during peripregnancy and emphasize that maintaining normal Hcy levels not only benefits maternal health but also has long-term beneficial effects on offspring health.

### Limitations and future perspectives

While this study establishes a link between maternal HHcy and genetic ovarian dysfunction, several limitations should be acknowledged. First, our methionine-enriched diet model cannot fully dissociate HHcy-specific effects from broader one-carbon metabolic perturbations, and the continuous exposure window prevents identification of the most vulnerable developmental stages. Second, our whole-ovary phenotypic analyses lack the mechanistic depth—at both the molecular and cell type-specific levels—to determine whether HHcy primarily targets oocytes, pregranulosa cells, or their synergistic interaction, and through which pathways (e.g., epigenetic, oxidative, and apoptotic). Third, the ovarian dysfunction observed in F1 offspring may have resulted from direct *in utero* exposure rather than true epigenetic inheritance, necessitating multigenerational studies to confirm transgenerational transmission. Addressing these limitations represents key directions for our ongoing research, such as integrating stage-specific exposure models, single-cell multi-omics, and conditional knockout strategies to precisely delineate how HHcy contributes to follicle overactivation and female fertility decline.

## Resource availability

### Lead contact

Further information and requests for resources and reagents should be directed to and will be fulfilled by the lead contact, Rong Hu (hur@nyfy.com.cn).

### Materials availability

This study did not generate any new material.

### Data and code availability

• All raw RNA-seq data have been deposited in GEO under accession number GSE322825 and are listed in the key resources table.
• This paper does not report original code.
• The lead contact will provide any additional information required for reanalysis of the data presented in this study upon request.

## Acknowledgments

We would like to express our sincere gratitude to the Ningxia Key Laboratory of Clinical Pathogens, Stem Cell Research Institute, 10.13039/501100025377General Hospital of Ningxia Medical University, Ningxia, China, for their support. We extend our sincere gratitude to the Medical Science and Technology Research Center of Ningxia Medical University, Ningxia, China, for their valuable support of this research. Special thanks go to Chao Wang, the head of the State Key Laboratory of Agrobiotechnology, College of Biological Sciences, China Agricultural University, Beijing, China, whose invaluable assistance with laboratory equipment and collaboration was essential to the progress of this research. We are also grateful to Yang T. (East China University of Political Science and Law, Shanghai, China) for participating in sample collection and testing during the holiday break. Finally, we would like to express our heartfelt thanks to all the participants in our study for their cooperation and contribution. Funding sources include the Natural Sciences Foundation of Ningxia grant number 2022AAC02060 to R.H., the 10.13039/501100001809National Natural Science Foundation of China award number 82160293 to R.H., the Key Development and Research Program of Ningxia grant number 2022CMG02028 to R.H., Central Special Program for Guiding Local Science and Technology Development (2024FRD05140) to R.H., the Key Research and Development Program Project of Ningxia Hui Autonomous Region (2025BEG02003) to R.H., the 10.13039/501100004772Ningxia Natural Science Foundation grant number 2023AAC03674 to J.M., the 10.13039/501100004772Ningxia Natural Science Foundation grant number 2023AAC03621 to J.W., and the Key Development and Research Program of Ningxia grant number 2023BEG03043 to J.W.

## Author contributions

J.G. played a key role in carrying out the experiments, collecting and analyzing the data, interpreting the results, and writing the manuscript; L.W. focused on organizing and managing the data, creating visual representations of the findings, and making revisions to the manuscript; J.M. and J.L. were responsible for overseeing the execution of the experiments and ensuring that the results were verified accurately; Y.W. and J.W. designed the research methodology and techniques and managed and coordinated the overall project. Rong Hu was in charge of the study’s overall conceptualization and design, secured the necessary grant funding, and supervised the project. All the authors carefully reviewed and approved the final version of the revised manuscript.

## Declaration of interests

The authors of this article declare that none of the work presented in this research could have been affected by any known conflicts involving financial interest or intimate relationships.

## STAR★Methods

### Key resources table

**Table:** 

REAGENT or RESOURCE	SOURCE	IDENTIFIER
**Antibodies**		

MVH/DDX4	Abcam	Cat# ab27591; RRID: AB_11139638
mTOR	CST	Cat# 2983; RRID: AB_2105622
*p*-mTOR	Abcam	Cat# sc-293133; RRID: AB_2861149
FOXO3	CST	Cat# 12829; RRID: AB_2636990
*p*-FOXO3	CST	Cat# 9466; RRID: AB_2106674
AKT	CST	Cat# 4691; RRID: AB_915783
*p*-AKT	Nature Biosciences	Cat# A52820; RRID: AB_2936343
RPS6	CST	Cat# 2317
*p*-RPS6	CST	Cat# 5364
Tubulin	Abcam	Cat# ab18207; RRID: AB_444319
ΓH2A.X	Abcam	Cat# ab81299; RRID: AB_1640564
β-Actin	Abmart	Cat# T40104; RRID: AB_2936320

**Chemicals, peptides, and recombinant proteins**		

Serum gonadotropin for injection (PMSG)	Ningbo Second Hormone Company	Cat#110254564
Human chorionic gonadotropin (hCG)	Ningbo Second Hormone Company	Cat#110251282
Hyaluronidase digestion	Nanjing Aibei Biotechnology Co., Ltd.	Cat#M2215
Hoechst 33342	Sigma‒Aldrich	Cat#B2261
4%Paraformaldehyde	Beyotime	Cat#P0099
M2 medium	Sigma‒Aldrich	Cat#M7167
M16 medium	Sigma‒Aldrich	Cat#M7292
Mineral oil	Sigma‒Aldrich	Cat#M5310
Masson trichrome staining	Beyotime	Cat#C0189S
MitoProbe JC-1 Assay Kit	KeyGEN BioTECH,	Cat#KGA1904-100
HTF medium	Nanjing Aibei Biotechnology Co., Ltd.	Cat#M1135
KSOM medium	Nanjing Aibei Biotechnology Co., Ltd.	Cat#M1435

**Deposited data**		

Raw RNA-seq data	This paper	GEO:GSE322825 (https://www.ncbi.nlm.nih.gov/geo/query/acc.cgi?acc=GSE322825)

**Software and algorithms**		

GraphPad Prism software	N/A	GraphPad Prism 10
ImageJ	N/A	ImageJ (https://imagej.net/)
Oebiotech	N/A	https://cloud.oebiotech.cn/#/home

### Experimental model and study participant details

#### Animals

The mice used in this study were C57BL/6 J mice sourced from the Animal Experiment Center of Ningxia Medical University and were bred in accordance with the guidelines of the university’s Animal Care Committee. C57BL/6 J is a widely used inbred strain known for its genetic stability and suitability for various studies, including immunology, neurobiology, and metabolic diseases. The colony was maintained through sibling mating to preserve genetic homogeneity. All the mice were housed in a specific pathogen-free (SPF) environment under the following controlled conditions: a temperature range of 24°C–26°C, a relative humidity of 55% ± 10%, a 12-h light/dark cycle, and *ad libitum* access to food and water. The animal care and experimental procedures were approved by the Institutional Animal Care and Use Committee (IACUC-2024-012) and adhered to the principles of laboratory animal welfare and ethics.

To establish a robust hyperhomocysteinemia (HHcy) model, three-week-old female mice were randomly divided into control (0.67% methionine) and high-methionine groups.^21^ The mice in the high-methionine group were fed a special diet containing 3% methionine, while those in the control group received a standard diet supplemented with 0.67% methionine (diets were purchased from Xiao Shu You Tai Company, Beijing, China). A plasma homocysteine (Hcy) cut-off of 15 μmol/L was used as the validation threshold, substantially higher than both the normal murine range (3–8 μmol/L)^64^ and the established HHcy threshold (≥10 μmol/L),^65^ to induce a pronounced and consistent HHcy phenotype. Preliminary experiments confirmed the model stability: after 6 weeks on the high-methionine diet, all the mice in the model group achieved serum Hcy concentrations exceeding 15 μmol/L (Figure S1). Following the 6-week feeding period, females were paired with fertile males at a 2:1 ratio. Plug-positive females were designated E0.5; pups were designated Pd0 at birth. The offspring were labeled F1-Control or F1-HHcy accordingly.

### Method details

#### Growth and development evaluation

F1 survival and weight gain were monitored after birth. The number of F1 offspring was recorded daily to track survival, and weight gain was measured and recorded weekly until 10 weeks of age. Anogenital distance (AGD) was assessed at postnatal day 21 (Pd21) by gently restraining the mice to ensure stability and full exposure of the anogenital region. For female mice, the vagina served as the genital marker. The distance from the anal verge to the genitalia was measured accurately with a caliper and recorded. Starting from Pd21, F1 mice were observed for vaginal opening, a key marker of sexual maturation. Observations were made at a fixed time each morning to accurately record the onset of vaginal opening until the first occurrence was noted for each mouse.

#### Histological analysis

The ovaries were collected, fixed overnight in 4% paraformaldehyde (Beyotime, P0099), dehydrated through a series of graded ethanol solutions, and embedded in paraffin. Thin sections (5 μm) were then cut. For follicle counting, all sections from each ovary were stained with hematoxylin (Solarbio, G1140), and the number of follicles at different developmental stages was determined on the basis of the criteria established by Pedersen and Peters.

For immunohistochemical (IHC) staining, the sections were treated sequentially with xylene, graded ethanol, sodium citrate buffer, and 3% H2O2. The sections were incubated overnight at 4°C with the primary antibody (Table S1), followed by incubation with the secondary antibody (Table S1) at 37°C for 60 min. Color development was performed using a DAB peroxidase substrate kit (Nakasugi Gold Bridge). Positive cells were counted using ImageJ (NIH, USA).

For immunofluorescence (IF) staining, tissue sections were treated with xylene, graded ethanol, and sodium citrate buffer before being blocked with 10% donkey serum (Yeasen, 36136ES60) for 60 min. The sections were incubated overnight at 4°C with the primary antibody (Table S1), followed by costaining with the secondary antibody and Hoechst (Beyotime, C1029) (Table S1) at 37°C for 60 min. Imaging was performed using a laser confocal microscope (Nikon A1). Positive cell counts and fluorescence intensity were analyzed using ImageJ (NIH, USA).

#### RNA-seq and analysis

RNA extraction, library construction, and transcriptome sequencing were carried out by Shanghai Ouyi Biotechnology Co., Ltd. (Shanghai, China). The sequencing data were subsequently analyzed using the Ouyi BioCloud platform (https://cloud.oebiotech.cn/#/home).

#### Oocyte collection and staining

F1 mice were injected intraperitoneally with PMSG, followed after 46 h by treatment with HCG. After 15 h, the cumulus–oocyte complexes (COCs) were retrieved from the oviducts and digested with hyaluronidase (AibeiBio, M2215) to isolate MII (Metaphase II) oocytes for subsequent staining. The oocytes were fixed in 3.7% paraformaldehyde (Beyotime, P0099) and blocked for 1 h in PBS containing 1% bovine serum albumin (Sigma, V900933). They were then incubated overnight with primary antibodies (Table S1) diluted in blocking solution. Afterward, the oocytes were incubated with secondary antibodies (Table S1) for 1 h in the dark. The nuclei were costained with Hoechst 33342 (Beyotime, C1029) and observed under a laser scanning confocal microscope (Nikon A1).

ROS levels in the oocytes were measured using the DCFH-DA (2,7-dichlorofluorescein diacetate) probe (Nanjing Jiancheng Bioengineering Institute, E004-1-1). Oocytes were incubated with the DCFH-DA working solution at 37°C for 20 min, washed twice with PBS, and imaged using a Nikon A1 laser confocal microscope.

The mitochondrial membrane potential was assessed using a Mito Probe JC-1 Assay Kit (Key GEN Bio TECH, KGA1904-100). The JC-1 working solution was prepared in M16 medium, and the oocytes were incubated with it for 20 min at 37°C and 5% CO2. Afterward, they were washed twice with buffer and immediately imaged using a Nikon A1 laser confocal microscope.

#### Breeding test

Twelve 8-week-old female offspring were paired with fertile adult males at a 2:1 ratio. Pregnancy was confirmed, and the females were kept separate from the males until delivery. The females did not receive treatments with pregnant mare serum gonadotropin (PMSG) or human chorionic gonadotropin (HCG). The number of litters was recorded on the day of birth, and the surviving pups were counted one week later.

#### Serum Hcy and hormone level determination

Serum samples were collected during the diestrus stage and confirmed by daily vaginal cytology for at least two consecutive estrous cycles prior to sacrifice. The mice were intraperitoneally injected with 4% chloral hydrate, and blood was collected from the eyeballs. The blood was left at room temperature for 2 h and then centrifuged at 3,000 rpm for 10 min to obtain the supernatant. Serum homocysteine (Hcy) concentration was measured using an Hcy enzyme-linked immunosorbent assay (ELISA) kit (JL-T1120; JONLN, China). Serum levels of estradiol (E2; JL11790-96 T; Laibio, China), follicle-stimulating hormone (FSH; JL20476-96 T; Laibio, China), anti-Müllerian hormone (AMH; JL10239-96 T; Laibio, China), luteinizing hormone (LH; JL16114-96 T; Laibio, China), and testosterone (T; JL10705-96 T; Laibio, China) were also determined using ELISA kits.

#### Western blotting

Total ovarian protein was extracted using lysis and extraction buffers (Goldbio, KGP250, Nanjing, China). The proteins were separated by 10% SDS‒PAGE at 80–120 V and then transferred to PVDF membranes (Millipore, ISEQ00010, USA). The membranes were blocked with Rapid Closure Buffer (Beyotime, P0231) for 15 min and incubated with primary antibody overnight at 4°C. Afterward, the membranes were incubated with the secondary antibody for 1 h at room temperature. Protein signals were detected using an enhanced chemiluminescence (ECL) kit (KeyGEN BioTECH, KGC4602-200), and grayscale quantification was performed using ImageJ (NIH, USA).

### Quantification and statistical analysis

All experiments were conducted at least three times, and the data are presented as medians (interquartile ranges) or medians (ranges) because of the small sample size (n = 3–12). Comparisons between the F1-Control and F1-HHcy groups were performed using the Mann‒Whitney U test (nonparametric test) with GraphPad Prism 10.1 software (GraphPad Software, San Diego, CA, USA). Statistical significance was set at ∗*p* < 0.05, ∗∗*p* < 0.01, or ∗∗∗*p* < 0.001.
