# Supplementary material for: Maternal hyperhomocysteinemia compromises female offspring fertility through overactivation of primordial follicles
Source: iScience. 2026 Jun 19;29(7):116393. doi: 10.1016/j.isci.2026.116393 (PMC13315451; doi:10.1016/j.isci.2026.116393)
Supplement: Document S1. Figures S1–S6 [file mmc1.pdf]

iScience, Volume *29*

Supplemental information

Maternal hyperhomocysteinemia compromises female offspring fertility through overactivation of primordial follicles

Jinmei Gao, Lu Wang, Jie Ma, Jialing Li, Yajie Wang, Jinfang Wang, and Rong Hu

Supplemental Figures

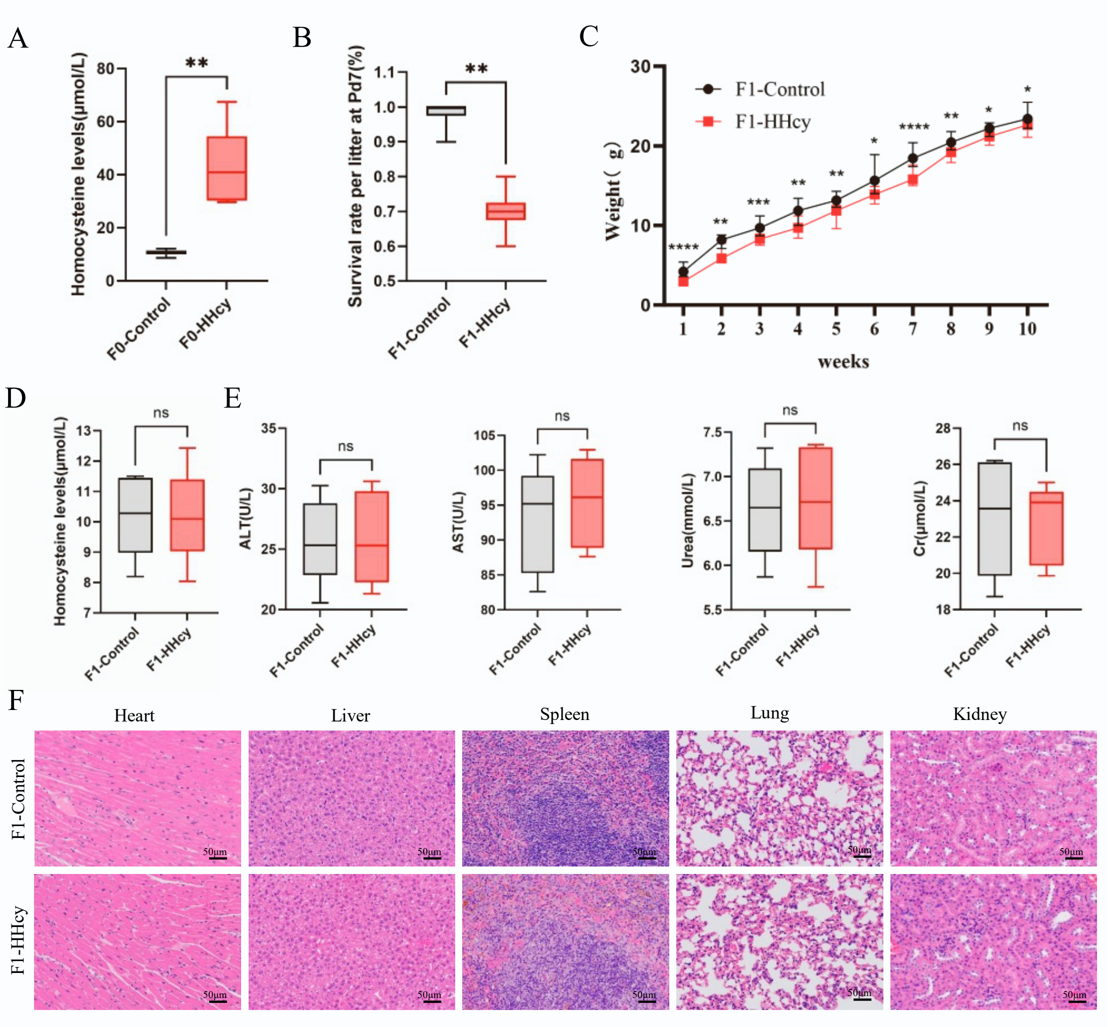

Supplementary Figure 1: Maternal HHcy decreased survival rates and slowed growth in F1 offspring but did not impact organ function. A. Determination of serum Hcy levels in F0 model female mice (n=6). B. Survival rate of F1 mice 7 days after birth (n=6). C. Monitoring body weight gain in female F1 mice (n=6). D. Serum Hcy levels in adult F1 female mice (n=6). E. Serum levels of ALT, AST, urea, and creatinine in adult F1 female mice (n=6). F. Histological morphology of the heart, liver, spleen, lungs, and kidneys of adult F1 female mice (n=6), scale=50μm. The data are presented as box plots (median with interquartile range), and statistical analysis was performed using the Mann-Whitney U test. *P<0.05, **P<0.01, ***P<0.001, ****P<0.0001.

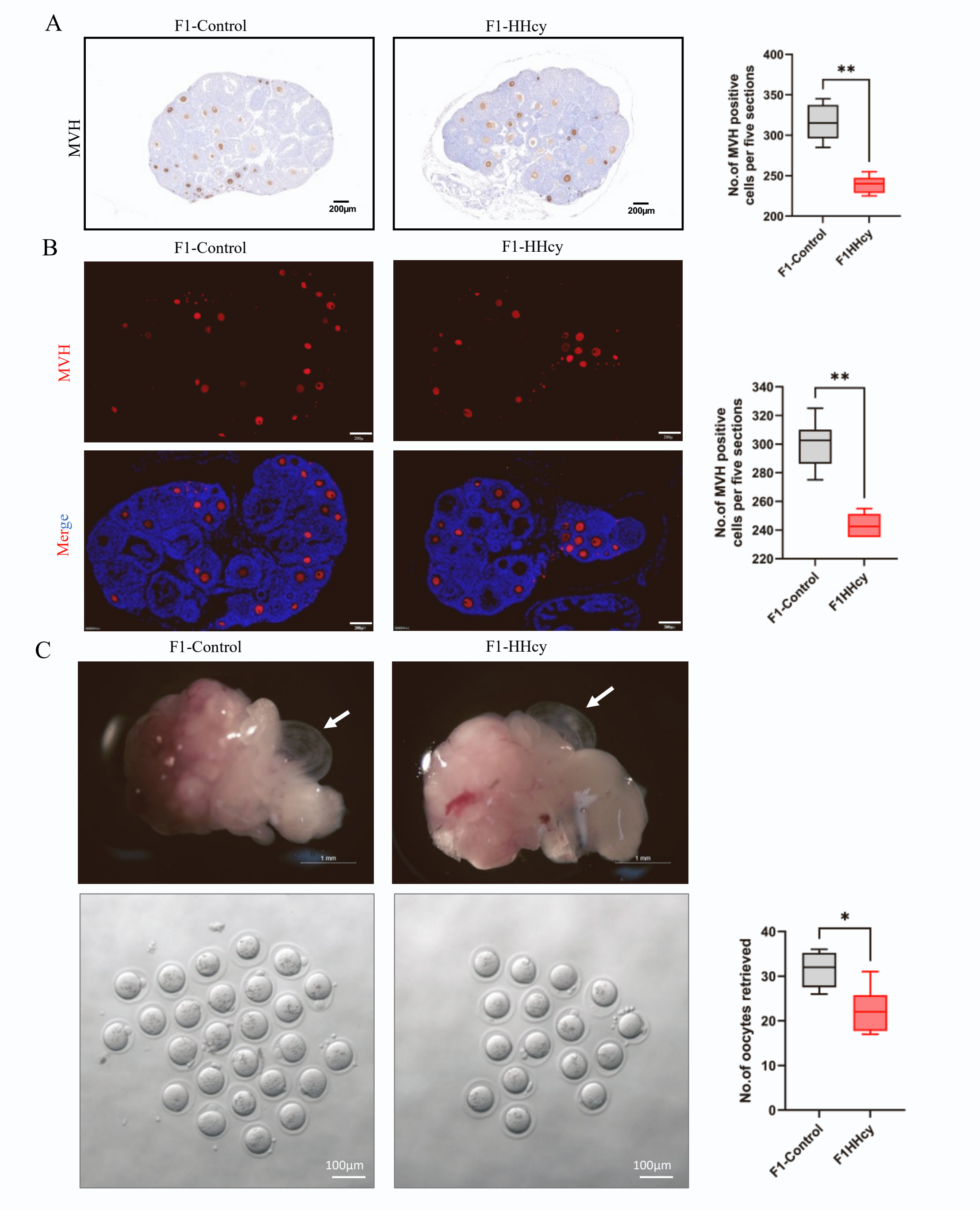

Supplementary Figure 2: Maternal HHcy impairs the ovarian reserve in F1 female mice. A. Immunohistochemical staining of MVH in ovarian tissue from F1 female mice at Pd21 (n=3), scale=200μm. B. Immunofluorescence staining of MVH in ovarian tissue from F1 female mice at Pd21 (n=3), scale=200μm. C. Representative image of the ampulla after ovulation in Pd21 female mice, scale=1mm, representative images of MII oocytes, and quantification of MII oocytes in each group (n=6), scale=100μm. The data are presented as box plots (median with interquartile range), and statistical analysis was performed using the Mann-Whitney U test. *P<0.05, **P<0.01.

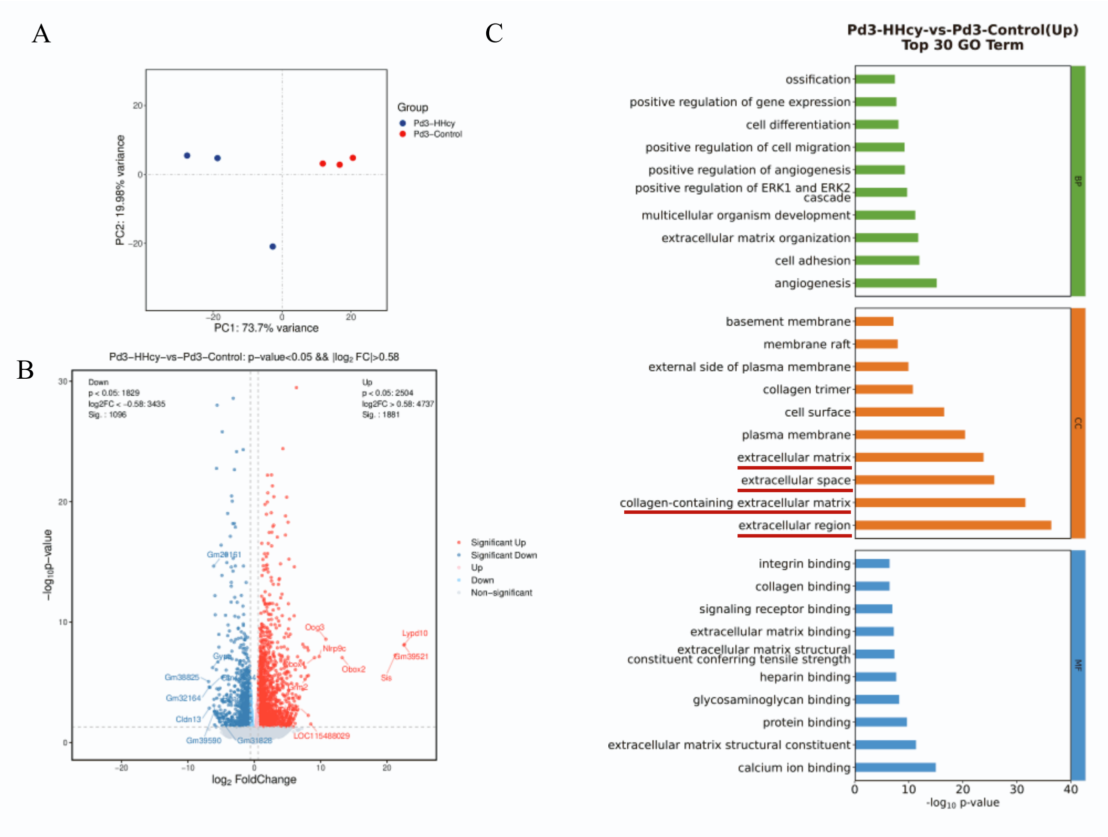

Supplementary Figure 3: Maternal HHcy leads to changes in transcript levels in the ovarian tissue of F1 on Pd3. A. PCA plot of the RNA-seq results of ovarian tissue on Pd3 in generation F1, indicating good sample consistency within the group. B. Volcano plot showing that 1881 genes were upregulated and 1096 genes were downregulated in the Pd3-HHcy group compared with the Pd3 control group. C. GO enrichment analysis of differentially expressed genes revealed that the most significant functional changes associated with CC (cellular component) included mainly the extracellular matrix, extracellular space, collagen-containing extracellular matrix, and extracellular region.

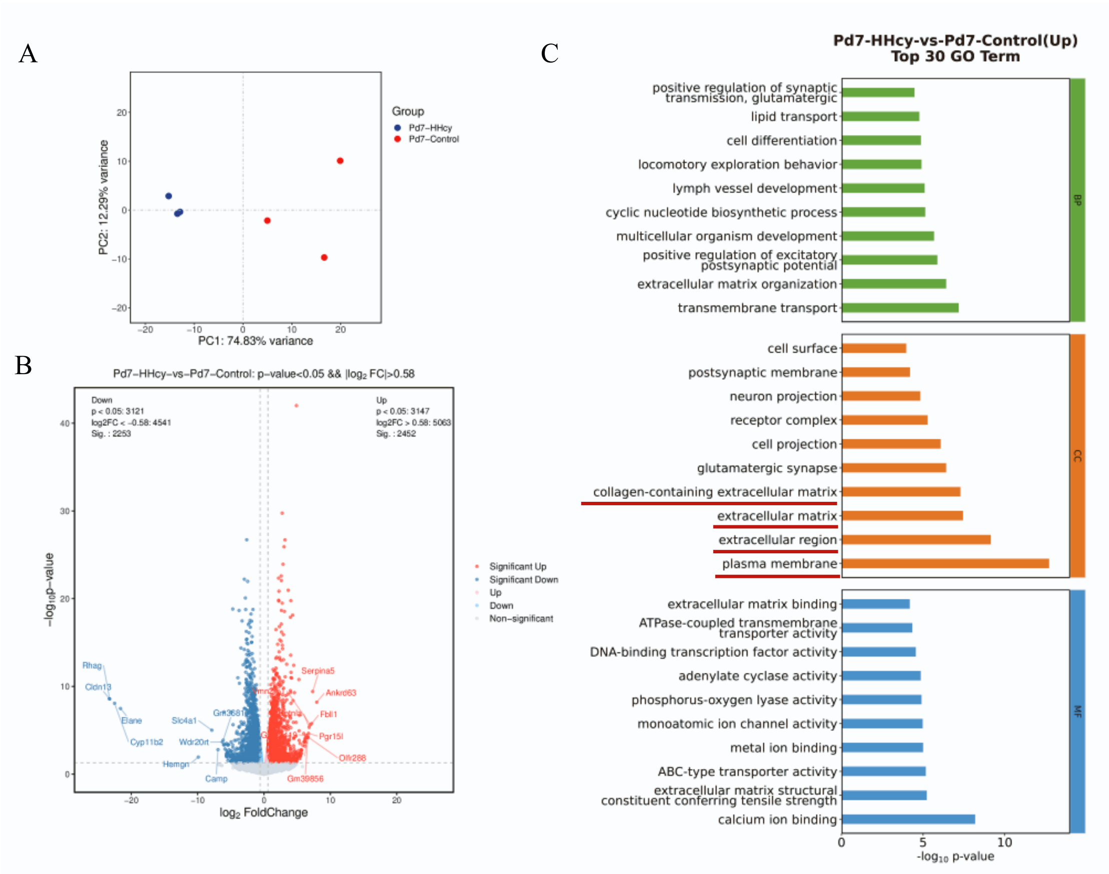

Supplementary Figure 4: Maternal HHcy induces changes in transcript levels in the ovarian tissue of F1 on Pd7. A. PCA plot of RNA-seq results of ovarian tissue on Pd7 in generation F1, indicating good sample consistency within the group. B. Volcano plot showing that 2452 genes were upregulated and 2253 genes were downregulated in the Pd7-HHcy group compared with the Pd7 control group. C. GO enrichment analysis of differentially expressed genes revealed that the most significant functional changes associated with CC (cellular component) included mainly those associated with the collagen-containing extracellular matrix, extracellular matrix, extracellular region, and plasma membrane.

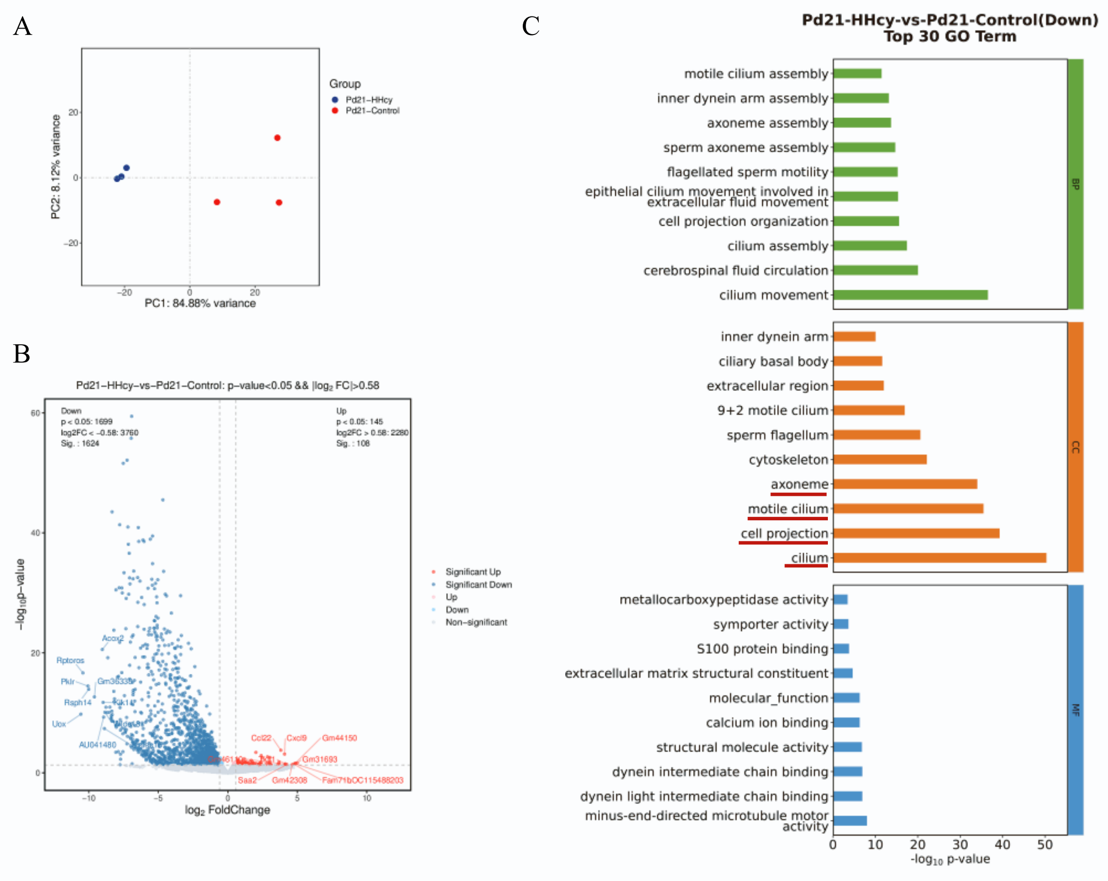

Supplementary Figure 5: Maternal HHcy causes changes in transcript levels in the ovarian tissue of F1 on Pd21. A. PCA plot of RNA-seq results of ovarian tissue on Pd21 in generation F1, indicating good sample consistency within the group. B. Volcano plot showing that 108 genes were upregulated and 1624 genes were downregulated in the Pd21-HHcy group compared with the Pd21-control group. C. GO enrichment analysis of differentially expressed genes revealed that the most significant functional changes associated with CC (cellular component) included mainly the axoneme, motile cilium, and cell projection cilium.

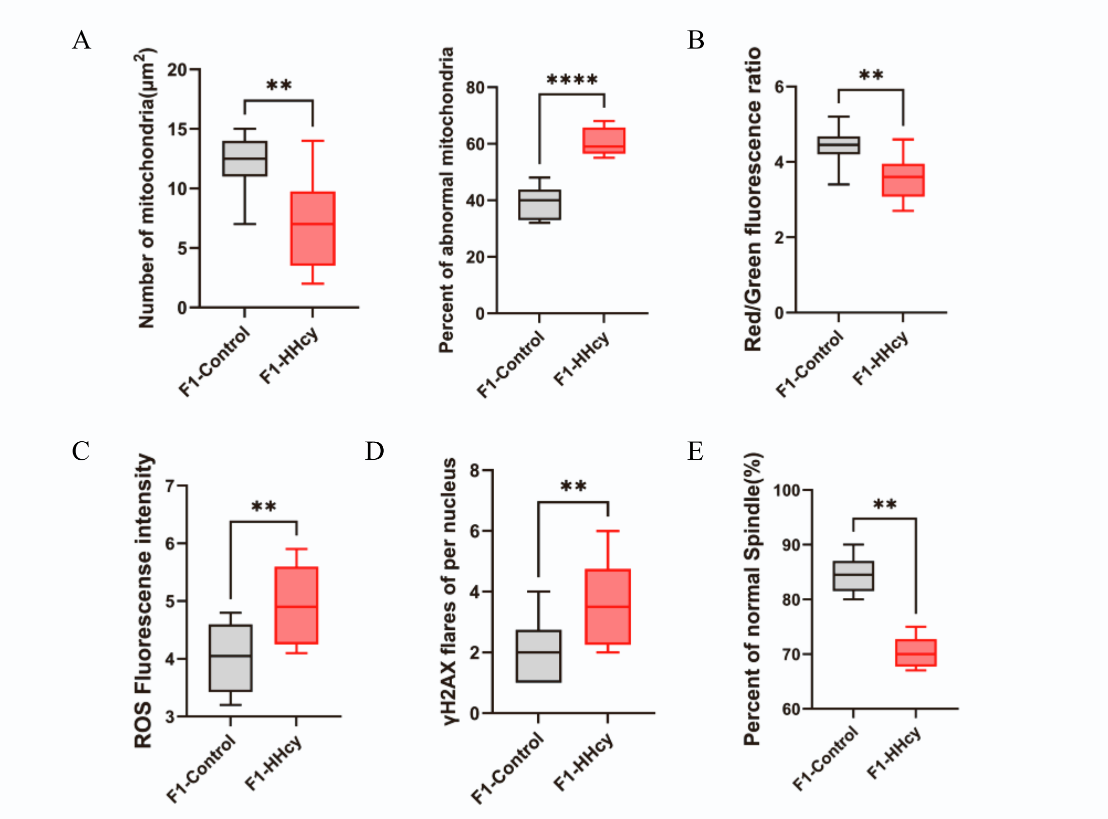

Supplementary Figure 6: Assessment of oocyte quality in F1 female mice. A. Quantification of the number of mitochondria in oocytes from F1 female mice (counting method: each oocyte was divided into four quadrants, the number of mitochondria in each quadrant was counted in 1 μm², and 3 oocytes were counted in each group), and the proportion of abnormal mitochondria in the oocytes was determined (n=3). B. Quantification of mitochondrial function in F1 female mouse oocytes using JC-1 staining (n=12). C. Quantification of oocyte ROS levels in F1 female mouse oocytes (n=12). D. Quantification of gamma-H2AX fluorescence foci in oocytes from F1 female mice to assess DNA damage (n=12). E. Quantification of the proportion of normal spindle bodies by tubulin fluorescence staining of oocytes from F1 female mice (n=25). The data are presented as box plots (median with interquartile range), and statistical analysis was performed using the Mann-Whitney U test. *P<0.05, **P<0.01, ***P<0.001, ****P<0.0001.
